# A novel measure to characterise optimality of diameter relationships at retinal vascular bifurcations

**DOI:** 10.1016/j.artres.2010.06.003

**Published:** 2010-09

**Authors:** Nicholas W. Witt, Neil Chapman, Simon A. McG. Thom, Alice V. Stanton, Kim H. Parker, Alun D. Hughes

**Affiliations:** International Centre for Circulatory Health, NHLI Division, Faculty of Medicine, Imperial College London, 59 North Wharf Road, London W2 1LA, UK

**Keywords:** Retina, Microvascular diameter, Vascular bifurcation, Bifurcation optimality, Junction exponent

## Abstract

Conventionally, the relationship between parent and daughter vessels at vascular bifurcations has been expressed by the junction exponent (*x*), and deviations of this parameter from the optimal conditions predicted by Murray’s law (*x* = 3) have been shown to be associated with vascular disease. However, the junction exponent is normally calculated iteratively from diameter measurements, and Monte-Carlo simulation studies show the junction exponent to be biased in the presence of measurement noise.

We present an alternative parameter, referred to as optimality ratio, that is simpler to compute and also more robust in the presence of noise.

To demonstrate the sensitivity of the optimality ratio to alterations in topography of the retinal vascular network, we analysed the effect of inducing endothelial dysfunction by infusion of NG-monomethyl-l-arginine (l-NMMA), a nitric oxide synthase inhibitor, compared to placebo in a double-blind crossover study. The optimality ratio showed a significant increase (*p* = 0.03) during infusion of l-NMMA compared to placebo.

We propose that a measure of the extent of departure of optimality ratio from its optimal value of 2^−1/3^ may be a useful indicator of microvascular endothelial dysfunction *in vivo*.

## Introduction

The relationship between diameters of parent and daughter vessels at a bifurcation is conventionally described by the junction exponent (*x*), defined by the relationship

(1) $$d_{0}^{x}=d_{1}^{x}+d_{2}^{x}$$

where *d*_0_ is the diameter of the parent vessel and *d*_1_, *d*_2_ are the diameters of the daughter vessels. Murray’s law^1^ predicts that under conditions of optimum power loss in the bifurcation, the junction exponent is equal to 3. Sherman^2^ and LaBarbera^3^ reviewed data from a variety of other workers, and showed that with the exception of the very largest vessels, healthy arteries and veins appeared to follow Murray’s law closely. In contrast, diseased vessels exhibit deviations from the diameter relationship predicted by Murray’s law. Hutchins et al.^4^ found a reduction in junction exponent in diseased human coronary arteries compared to normal subjects. In the retinal arteries, Stanton et al.^5^ have shown a reduction in junction exponent with age, and Chapman et al.^6^ have found similar alterations to retinal bifurcation geometry in peripheral artery disease.

The junction exponent is a convenient mathematical description of the diameter relationships at bifurcations, and the expected behaviour of the junction exponent has been studied theoretically by optimisations based on alternative cost functions.^7^ Nevertheless, an experimentally calculated junction exponent cannot in general be related directly to fundamental hemodynamic or physiological characteristics. Due to the exponential formulation, it is usual to calculate the junction exponent from a set of diameter measurements by an iterative process or a look-up table, thus adding computational complexity. Furthermore, the junction exponent can be expected to suffer excessive bias and variability in the presence of noise in the measurements of individual vessel diameters.

Accordingly, we propose an alternative parameter to characterise the optimality of the relationship between vascular diameters at a bifurcation, referred to as the optimality ratio, which overcomes the drawbacks with the junction exponent.

We report here first on the derivation of the optimality ratio, and go on to describe Monte-Carlo simulations which demonstrate improved robustness to measurement noise on individual vessel measurements. We have also calculated the optimality ratio from retinal measurements made in a clinical study, thereby demonstrating the sensitivity and utility of the optimality ratio as a measure of arteriolar changes resulting from inhibition of nitric oxide (NO) synthase induced by infusion of NG-monomethyl-l-arginine (l-NMMA).

## Derivation of optimality ratio

For convenience, we define non-dimensional variants of the daughter diameters at a vascular bifurcation

(2) $$\zeta_{1}=\frac{d_{1}}{d_{0}},\;\zeta_{2}=\frac{d_{2}}{d_{0}}$$

Hence, from Eqs. (1) and (2) the definition of junction exponent may be re-stated as

(3) $$\zeta_{1}^{x}+\zeta_{2}^{x}=1$$

**Figure 1:**
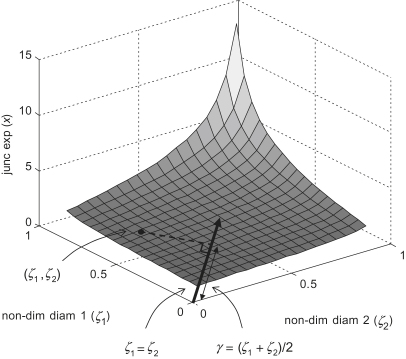
Junction exponent as a function of non-dimensional daughter diameters *ζ*_1_ and *ζ*_2_.

Insight may be gained into the behaviour of the junction exponent (*x*) by plotting it against the non-dimensional daughter diameters *ζ*_1_ and *ζ*_2_ as shown in Fig. 1. The line *ζ*_1_ = *ζ*_2_ represents the set of perfectly symmetrical bifurcations, and departures from this line are associated with increasing asymmetry. In general, the projection of a point in the *ζ*_1_, *ζ*_2_ plane, onto the line *ζ*_1_ = *ζ*_2_ represents the mean daughter diameter, non-dimensionalised by the parent diameter. We will refer to this as the mean diameter ratio

(4) $$\gamma =\frac{d_{1}+d_{2}}{2d_{0}}=\frac{\zeta_{1}+\zeta_{2}}{2}$$

An evident feature of the illustrated surface in Fig. 1 is that the relationship between mean diameter ratio (*γ*) and junction exponent (*x*) appears largely insensitive to bifurcation asymmetry. Accordingly, a parameter based on *γ* offers the prospect of a robust, statistically well-behaved alternative to the junction exponent. Detailed examination of plots of *γ* against *x* for a range of symmetrical and asymmetrical bifurcations reveal a small residual dependency on asymmetry concentrated in the region of optimality (*x* = 3). Since small deviations are of particular interest in this region, it is desirable to correct for this.

Conventionally, the asymmetry factor (*α*) of a bifurcation is defined^8^ as follows

(5) $$\alpha ={\left(\frac{d_{1}}{d_{2}}\right)}^{2}={\left(\frac{\zeta_{1}}{\zeta_{2}}\right)}^{2}\;\text{where}\;d_{1},d_{2}>0\;\text{and}\;d_{1}\geq d_{2}$$

so that a perfectly symmetrical bifurcation is described by *α* = 1.

By algebraic manipulation of Eqs. (3)–(5) it may be shown that for a general bifurcation

(6) $$\gamma =\frac{1+\alpha^{1/2}}{2{\left(1+\alpha^{x/2}\right)}^{1/x}}\text{.}$$

Under optimum conditions (*i.e. x* = 3) the value of mean non-dimensional daughter diameter is given by

(7) $$\gamma^{*}=\frac{1+\alpha^{1/2}}{2{\left(1+\alpha^{3/2}\right)}^{1/3}}$$

that is dependent only on asymmetry, and for a symmetrical bifurcation (*i.e. α* = 1) takes the constant value

(8) $$\gamma^{**}=\frac{1}{2^{1/3}}=0.7937.$$

We therefore propose a ratio to characterise the optimality of the diameter relationships at a bifurcation, that we refer to as the optimality ratio

(9) $$\Gamma_{\text{ratio}}=f\left(\alpha \right)\gamma$$

where the correction factor

(10) $$f\left(\alpha \right)=\frac{\gamma^{**}}{\gamma^{*}}=\frac{{\left\{4\left(1+\alpha^{3/2}\right)\right\}}^{1/3}}{\left(1+\alpha^{1/2}\right)}$$

adjusts the measured mean diameter ratio, to take account of the measured asymmetry, yielding the approximate value that would have been obtained for a symmetrical bifurcation. For a bifurcation obeying Murray’s law, the correction is exact.

By algebraic simplification of Eqs. (4), (9) and (10)

(11) $$\Gamma_{\text{ratio}}={\left(\frac{d_{1}^{3}+d_{2}^{3}}{2d_{0}^{3}}\right)}^{1/3}\text{.}$$

Thus, the optimality ratio (*Γ_ratio_*) exhibits the desirable property that under optimal conditions predicted by Murray’s law it equals the constant 2^−1/3^, given by Eq. (8), irrespective of asymmetry.

**Figure 2:**
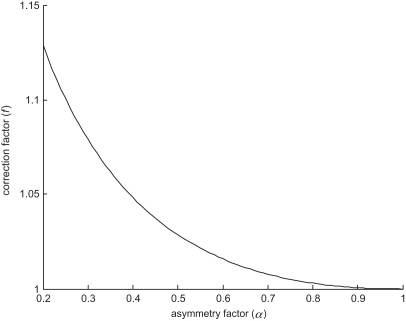
Dependence of correction factor on asymmetry.

The dependence of the correction factor *f*(*α*) on asymmetry factor is illustrated in Fig. 2. It can be seen that for reasonably symmetric bifurcations (*α* > 0.4), the correction factor has only a small effect on the overall non-dimensional daughter diameter, affecting it by less than 5%. However, for less symmetrical bifurcations, the effect of the correction factor is amplified, and reliable derivation of the corrected parameter may then be compromised by measurement noise, particularly in the diameter of the smaller daughter vessel. Therefore, wherever possible, we advocate exclusion of highly asymmetrical bifurcations (*α* < 0.4) from consideration of vessel diameter optimality. In practice this also avoids difficulties in measuring very small diameters.

**Figure 3:**
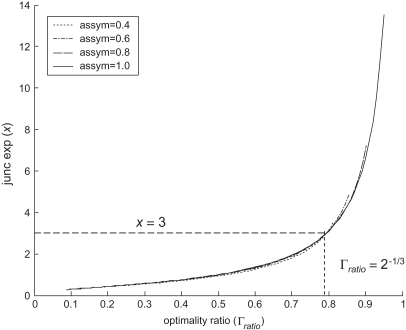
Junction exponent against optimality ratio.

The behaviour of the corrected optimality ratio may be illustrated by plotting the relationship with the junction exponent for various asymmetry factors, as shown in Fig. 3. This confirms that for a junction exponent of 3 the value of optimality ratio remains constant irrespective of asymmetry, and also shows only a negligible sensitivity to asymmetry away from optimal conditions.

For convenience we also define the optimality index

(12) $$\Gamma_{\text{ind}}=2^{1/3}\Gamma_{\text{ratio}}$$

that possesses similar characteristics to the optimality ratio, except that it takes the value of 1 under optimum conditions.

Finally, we define the optimality deviation, a measure of the extent of deviation from the optimum conditions

(13) $$\Gamma_{\text{dev}}=\left|\Gamma_{\text{ratio}}-\frac{1}{2^{1/3}}\right|\text{.}$$

## Robustness to measurement noise

Monte-Carlo simulations were performed to assess the robustness of the optimality ratio to measurement noise, in comparison with the junction exponent.

A symmetrical optimal bifurcation *(i.e. x* = 3 and *Γ*_ratio_ = 0.7937) was considered with the independent addition to each vessel of normally distributed measurement noise of zero mean, and standard deviation corresponding to 5, 10 and 15% of the vessel diameter. After 2000 trials, the mean biases in junction exponent and optimality ratio between ‘measured’ and true values were calculated, together with the standard deviation of differences. In order to allow meaningful comparison, the results from the junction exponent were scaled to units of optimality ratio through multiplication by Δ*Γ*_ratio_/Δ*x* = 0.06113, derived analytically from Eqs. (3), (4) and (9) at the point of optimality (*x* = 3). The results of this Monte-Carlo simulation are shown in Table 1.

It is evident from Table 1 that the optimality ratio is less severely affected by the simulated measurement noise than the junction exponent. For modest noise having standard deviation of up to 10% of the vessel diameter, the bias in the optimality ratio is less than one sixth of the value arising from the junction exponent. Furthermore, the scatter between ‘measured’ and true values of optimality ratio is about half that arising from the junction exponent.

A further trial was performed to evaluate how the variability and bias in the optimality ratio arising from measurement noise depend on the characteristics of the bifurcation under study. Table 2 shows the result of applying measurement noise of zero mean and standard deviation of 10% of vessel diameter to a selection of bifurcations characterised by optimality ratio and asymmetry factor.

These findings confirm that the optimality ratio remains well behaved through the range of bifurcations that are likely to be subject to clinical investigation.

## Application to clinical data

### Methods

To examine the clinical utility of the optimality ratio in characterising the diameter relationships at a vascular bifurcation, an analysis was undertaken of retinal microvascular data following induction of endothelial dysfunction using an NO-synthase inhibitor.

Data were obtained from a double-blind, randomised, placebo-controlled crossover trial involving six healthy normotensive (BP < 140/90 mmHg) male volunteers (age 22–32 years). All volunteers were screened for evidence of concomitant disease by physical examination, as well as biochemical and haematological tests. The study was carried out in accordance with the principles of the Declaration of Helsinki (1989) of the World Medical Association. All subjects gave written informed consent, and the local research ethics committee approved the study protocol.

Each subject received a 5-min intravenous infusion of l-NMMA (Clinalfa AG, Läufelfingen, Switzerland) (3mg kg^−1^ bodyweight in 0.9% saline) or matched placebo (20 ml 0.9% saline) in random order on 2 occasions separated by 2–7 days. On each occasion, subjects were required to abstain from alcohol, smoking and caffeine for 24 h prior to the study, and remained seated comfortably in a quiet room for 2 h during each phase of the study. The right pupil was dilated using a topical mydriatic (1% tropicamide; Chauvin Pharmaceuticals, Romford, England) and an intravenous cannula was sited in the dorsum of the left hand for infusion of study drugs. Brachial systolic and diastolic blood pressure and heart rate were measured at five-minute intervals for 20 min prior to, and for 1 h following infusion of the study drugs, using a validated automated oscillometric blood pressure monitor (Omron HEM-705 CP; Omron, Tokyo, Japan). Red-free 35 mm retinal photographs were taken on Ilford FP4 (125 ASA) photographic film (Ilford Imaging UK Ltd., Knutsford, England) using a fundus camera with a 30° field of view (Kowa FX-50R, Kowa, Tokyo, Japan). Duplicate views of the superior temporal quadrants were taken at 20 min and 10 min prior to infusion of the study drug and at 10, 20, 30 and 60 min following infusion.

Analysis of the retinal images was performed by a single trained observer using a validated, purpose built operator-directed image analysis package based on the Sliding Linear Regression Filter (SLRF) method of vessel diameter measurement, as described elsewhere.^9^ The photographic negatives were digitised using a Nikon 35 mm film scanner (LS-1000, Nikon, Tokyo, Japan), yielding images of 2800 × 2400 pixels in size. A pixel corresponded to an absolute distance of order 5 μm at the retina. In each subject, the same bifurcations were identified (5 per subject) and at each bifurcation the diameters of the parent vessel (*d*_0_), and daughter vessels (*d*_1_ and *d*_2_) were measured at each time point. All vessel diameters were measured in pixels, and no attempt was made to calculate actual arteriolar diameters due to uncertainties introduced by the refractive index of the eye and the distance between the retina and the camera lens.

Optimality ratio was calculated at each bifurcation in accordance with Eq. (11), and the median difference from baseline calculated at each time point within each subject. Data are presented as mean (SD) and statistical comparison of placebo vs. l-NMMA was performed using linear mixed models in Stata 11.0 to take account of repeated measures,^10^ with *p* < 0.05 being considered significant.

## Results

**Figure 4:**
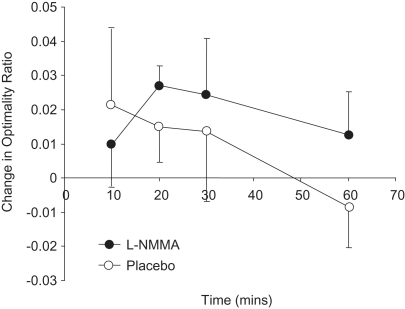
Change in optimality ratio (mean (SD)) during active and placebo phases of trial.

Baseline variables did not differ significantly between measurements made on the occasions of placebo and l-NMMA infusions (Table 3) and optimality ratios at baseline did not differ significantly from the predicted optimum value of 0.794. The optimality ratio of retinal bifurcations increased significantly (*p* = 0.03) following infusion of l-NMMA compared with placebo (Fig. 4; Table 4). l-NMMA infusion also significantly increased systolic (*p* < 0.001) and diastolic BP (*p* < 0.001) and reduced retinal arteriolar diameter (*p* = 0.03) (Table 4). Differences in heart rate and bifurcation angle between l-NMMA and placebo were not statistically significant. There was no significant statistical interaction between the reduction in retinal arteriolar diameter and bifurcation order suggesting that l-NMMA induces a generalized vasoconstriction in arterioles up to 5th order.

## Discussion

Previous studies implicate NO produced by the endothelium in maintaining the optimal configuration of vascular networks.^11–13^ In isolated rabbit ear preparations with unimpaired endothelial function, junction exponents remained close to theoretical optimum values even when preparations were vasoconstricted by serotonin. In contrast, when NO was inhibited by haemoglobin, vasoconstriction was associated with marked deviation of junction exponents from the optimum.

In the present study, infusion of l-NMMA, an inhibitor of NO synthase, resulted in a rise in blood pressure compared to placebo, consistent with previous studies that showed a rise in systemic vascular resistance following systemic infusions of l-NMMA.^14,15^ Two previous studies, one using fluorescein angiograms the other red-free images^5,16^ have both reported no difference in junction exponents between normotensive and hypertensive subjects, although ageing was associated with a decline in junction exponents.^5^ Accordingly, the increase in optimality ratio observed following infusion of l-NMMA appears most likely to have arisen from inhibition of NO synthase rather than being secondary to elevation of systemic blood pressure.

These data are consistent with previous *in vitro* observations, and indicate that optimality ratio is altered by inhibition of NO synthase through l-NMMA infusion. Furthermore, it supports the hypothesis that NO is important for the maintenance of optimal arterial topography *in vivo* in man.

## Conclusions

We have derived a parameter, referred to as the optimality ratio, aimed at characterising the diameter relationships at a vascular bifurcation. This parameter is based on the mean non-dimensional daughter diameter, but includes a correction factor to reduce the effect of bifurcation asymmetry. At an optimum bifurcation in accordance with Murray’s law (*i.e.* junction exponent = 3) the optimality ratio adopts a constant value of 2^−1/3^, and rises monotonically with increasing junction exponent. The optimality ratio can be readily calculated directly from measurements of vessel diameter, in contrast to the junction exponent which generally requires an iterative solution, or else cumbersome look-up tables.

Monte-Carlo simulation studies demonstrate that the optimality ratio also offers improved robustness, compared to the junction exponent, in the presence of measurement noise in individual vessels. For measurement noise having standard deviation up to 10% of vessel diameter, bias in optimality ratio was less than one sixth of that in junction exponent, and scatter in the region of one half, after adjustment for relative scaling.

In data obtained from a double-blind placebo-controlled clinical investigation, infusion of l-NMMA, a NO-synthase inhibitor, was shown to increase significantly the optimality ratio of retinal vessels, compared to placebo. This supports the hypothesis that NO is important for the maintenance of optimal arterial topography in man. Moreover, we propose that a measure of the extent of departure of optimality ratio from its optimal value of 2^−1/3^, may be a useful indicator of microvascular endothelial dysfunction *in vivo*.

## Sources of funding

This work was supported in part by a project grant from the Wellcome Trust (UK). NC, SAMcGT and ADH also received support from the National Institute for Health Research (NIHR) and NW and KHP received support from the Foundation for Circulatory Health (FCH). The sponsors had no involvement in the design or conduct of the study.

## Figures and Tables

**Table 1:** Effect of measurement noise on junction exponent and optimality ratio.

SD of measurement noise (% of vessel diameter)	Mean bias (SD)	
Units of optimality ratio	
From junction exponent	From optimality ratio
5	0.019 (0.087)	0.0030 (0.049)
10	0.060 (0.23)	0.0089 (0.10)
15	0.042 (0.32)	0.024 (0.16)

**Table 2:** Results of Monte-Carlo simulation of the effect of measurement noise at different asymmetry factors (*α*) on optimality ratio (*Γ*_ratio_).

*α*	Mean bias (SD)				
*Γ*_ratio_ = 0.6	*Γ*_ratio_ = 0.7	*Γ*_ratio_ = 0.8	*Γ*_ratio_ = 0.9	*Γ*_ratio_ = 1.0
1.0	0.008 (0.075)	0.009 (0.089)	0.014 (0.106)	0.014 (0.114)	0.018 (0.128)
0.8	0.008 (0.077)	0.010 (0.090)	0.017 (0.104)	0.011 (0.113)	0.017 (0.126)
0.6	0.008 (0.077)	0.009 (0.093)	0.012 (0.102)	0.011 (0.119)	0.015 (0.130)
0.4	0.011 (0.082)	0.009 (0.094)	0.009 (0.103)	0.015 (0.121)	0.016 (0.135)

**Table 3:** Baseline measurements in placebo and l-NMMA phases.

	Placebo	l-NMMA
Systolic BP, mmHg	129 (11)	127 (14)
Diastolic BP, mmHg	69 (9)	72 (9)
Heart rate, bpm	68 (7)	68 (11)
Diameter *d*_0,_ pixels	28.3 (3.1)	27.9 (3.8)
Bifurcation angle, degrees	77 (10)	79 (9)
Optimality ratios	0.784 (0.006)	0.795 (0.021)

Data are means (SD).

**Table 4:** Effect on measured variables of l-NMMA compared with placebo.

	Marginal effects (placebo–l-NMMA)	*p* Value
Systolic BP, mmHg	9.0 (4.1, 13.8)	< 0.001
Diastolic BP, mmHg	4.5 (2.3, 6.6)	< 0.001
Heart rate, bpm	−1.2 (−3.6, 1.3)	0.4
Diameter *d*_0,_ pixels	−0.72 (−1.38, −0.06)	0.03
Bifurcation angle, degrees	−1.1 (2.8, 0.7)	0.2
Optimality ratios	0.022 (0.002, 0.043)	0.03

Data are marginal means (95% confidence intervals).
